# The structure of the *Caenorhabditis elegans* TMC-2 complex suggests roles of lipid-mediated subunit contacts in mechanosensory transduction

**DOI:** 10.1073/pnas.2314096121

**Published:** 2024-02-14

**Authors:** Sarah Clark, Hanbin Jeong, Rich Posert, April Goehring, Eric Gouaux

**Affiliations:** ^a^Vollum Institute, Oregon Health and Science University, Portland, OR 97239; ^b^HHMI, Oregon Health and Science University, Portland, OR 97239

**Keywords:** native membrane protein complex, mechanosensory transduction complex, transmembrane channel-like protein

## Abstract

Mechanotransduction is the process by which a mechanical force, such as touch, is converted into an electrical signal. Transmembrane channel-like (TMC) proteins are an evolutionarily conserved family of membrane proteins whose function has been linked to a variety of mechanosensory processes, including hearing and balance sensation in vertebrates and locomotion in *Drosophila*. TMC1 and TMC2 are components of ion channel complexes, but the molecular features that tune these complexes to diverse mechanical stimuli are unknown. *Caenorhabditis elegans* express two TMC homologs, TMC-1 and TMC-2, both of which are the likely pore-forming subunits of mechanosensitive ion channels but differ in their expression pattern and functional role in the worm. Here, we present the single-particle cryo-electron microscopy structure of the native TMC-2 complex isolated from *C. elegans*. The complex is composed of two copies of the pore-forming TMC-2 subunit, the calcium and integrin binding protein CALM-1 and the transmembrane inner ear protein TMIE. Comparison of the TMC-2 complex to the recently published cryo-EM structure of the *C. elegans* TMC-1 complex highlights conserved protein–lipid interactions, as well as a π-helical structural motif in the pore-forming helices, that together suggest a mechanism for TMC-mediated mechanosensory transduction.

## Significance

One mechanism by which organisms sense their environment is through the perception of mechanical stimuli such as sound, touch, and vibration. Transmembrane channel-like (TMC) proteins are ion channels whose function has been linked to a variety of mechanosensitive processes, including hearing and balance in vertebrates and touch sensation in worms. The molecular mechanisms by which TMCs respond to mechanical stimuli are unknown. Here, we present the structure of the TMC-2 complex isolated from worms. Comparison of the TMC-2 complex to the recently solved structure of the worm TMC-1 complex highlights common structural features that are likely important for sensing mechanical stimuli yet also illuminates key differences that may explain the distinct functional roles of TMC-1 and TMC-2 in the worm.

Living organisms hear, sense touch, and perceive balance through mechanotransduction, the conversion of a mechanical force into an electrical signal. The primary sensors that mediate rapid responses to mechanical stimuli are mechanosensitive ion channels (1). Transmembrane channel-like (TMC) proteins are a family of ion channels whose function has been linked to a wide variety of mechanosensitive processes, including hearing in vertebrates (2, 3), water flow detection in zebrafish (4–6), and food texture sensation in *Drosophila* (7). Even within the same organism, TMC paralogs respond to different mechanical stimuli (8) and exhibit alternate responses to the same stimuli (2, 9). The features which tune homologous TMC proteins to diverse mechanical stimuli are unknown.

TMC proteins are conserved from *Caenorhabditis elegans* to humans (10, 11). Vertebrates express eight TMC paralogs, of which TMC1 and TMC2 are important for the sensations of hearing and balance (11). TMC1 and TMC2 are the pore-forming subunits of the mechanoelectrical transduction complexes that are located in auditory and vestibular hair cells of the inner ear (12, 13). Although mammalian auditory hair cells express both TMC1 and TMC2, the expression patterns differ during development, and they exhibit differences in current amplitude (2) and small molecule permeability (9). The six additional vertebrate TMC proteins (TMC3 to TMC8) are localized to various tissues, including the brain and colon (10, 11). Dysregulation of TMCs 4 to 8 have been linked to diverse human cancers (14, 15) and TMCs have been highlighted as promising biomarkers for cancer prognosis and potential therapeutic targets (15). However, the function of TMCs 3 to 8 remains unknown.

Invertebrates also express orthologs of TMC proteins, many of which have been implicated in mechanosensory processes. *Drosophila* express a single TMC ortholog that is important for larval locomotion and food texture sensation (7, 16). The *C. elegans* genome encodes two *tmc* genes, *tmc-1* and *tmc-2*, both of which encode proteins that are likely subunits of mechanosensitive ion channel complexes (8). The *C. elegans* TMC-1 protein has been implicated in a plethora of cellular processes, including sodium chemosensation (17), alkaline sensation (18), modulation of membrane excitability (19), touch sensation (8), metabolism (20), and regulation of GABA signaling (21). TMC-1 is localized to mechanosensory and chemosensory neurons, as well as body wall and vulval muscle (8). By contrast, *C. elegans* TMC-2 is localized exclusively to body wall and vulval muscles (8) and its role in worm physiology is less well explored. We recently solved the structure of the *C. elegans* TMC-1 complex, illuminating structural elements that are likely important for mechanosensation and providing insight into the structure of the vertebrate mechanosensory transduction (MT) complexes (22). Given the differences in function and expression pattern of *C. elegans* TMC-1 and TMC-2, we were curious how the architecture of the TMC-2 complex differs from that of TMC-1.

Here, we use cryo-electron microscopy (cryo-EM) to solve the structure of the native TMC-2 complex isolated from transgenic worms. Furthermore, to complement the structures of the TMC-1 and TMC-2 complexes, we used mass spectrometry to investigate whether additional proteins, such as ankyrin or UNC-44 in worms, are part of the TMC-2 complex. Previous research suggested that ankyrin couples TMC proteins to actin, functioning as an intracellular tether to relay mechanical force to the ion channel (8). The structure of the TMC-2 complex is strikingly similar to the TMC-1 complex, highlighting conserved structural features and protein–lipid interactions that shed light on how TMC complexes sense force. Further, the TMC-1 and TMC-2 complexes exhibit several key differences in the subunit composition and pore-lining amino acids that may render the complexes functionally distinct.

## Results

### Subunit Composition and Overall Architecture of the TMC-2 Complex.

To isolate the native TMC-2 complex from *C. elegans*, we employed the same strategy that was used for the TMC-1 complex (22, 23). In brief, we generated a transgenic worm line wherein the 3′ end of the TMC-2 coding sequence was tagged with a nucleic acid sequence encoding a mVenus fluorophore and a 3xFLAG tag (*tmc2::mVenus*). Using spectral confocal imaging, we characterized the *tmc2::mVenus* worm line and demonstrated that the TMC-2-mVenus protein is expressed in the body wall and vulval muscles (*SI Appendix*, Fig. S1*A*), in agreement with previous studies (8). The expression level of TMC-2 in *C. elegans* is roughly threefold higher than that of TMC-1, as assessed by fluorescent-detection size-exclusion chromatography (FSEC) experiments (24) using an equivalent number of *tmc1::mVenus* and *tmc2::mVenus* worms. To isolate the TMC-2 complex, we homogenized ~ 4 × 10^6^ *tmc2::mVenus* worms and performed affinity chromatography followed by fluorescent-detection size-exclusion chromatography (FSEC) (*SI Appendix*, Fig. S1*B*). The estimated molecular weight of the TMC-2 complex by FSEC was ~750 kDa, slightly smaller than that of the TMC-1 complex, but far larger than the predicted 328 kDa molecular weight of a TMC-2 dimer, suggesting that auxiliary proteins are bound to TMC-2.

Mass spectrometry analysis of the TMC-2 complex revealed a similar complex composition to TMC-1 (22), with one notable difference. The auxiliary proteins calcium and integrin binding CALM-1 and transmembrane inner ear protein TMIE were both identified as subunits of the TMC-2 complex by mass spectrometry (*SI Appendix*, Fig. S1*C*), in accordance with mass spectrometry results of the TMC-1 complex. Intriguingly, however, the TMC-1 auxiliary protein arrestin (ARRD-6), was not identified by mass spectrometry or by cryo-EM analysis of the TMC-2 complex. Because ARRD-6 interacts exclusively with CALM-1 and the intracellular leaflet of the membrane, and does not directly interact with TMC-1, it is unclear why ARRD-6 did not copurify with the TMC-2 complex. Differences in tissue localization between TMC-1 and TMC-2, specifically the absence of TMC-2 in sensory neurons, may explain the lack of TMC-2 association with ARRD-6. It is possible that ARRD-6 associates with TMC-1 complexes that are localized to neurons. The role of ARRD-6 in the function of the *C. elegans* TMC-1 channel is unknown and the vertebrate homolog, α-arrestin, is not a known component of the MT complex. Additional experiments are required to investigate how ARRD-6 plays a role in TMC-1 channel function. Further, we did not find evidence for TMC-2 association with UNC-44, the worm ortholog of mammalian ankyrin. UNC-44 has been reported to interact with CALM-1 and play a vital role in TMC-1- and TMC-2-mediated mechanosensation in worms (8), yet we did not detect UNC-44 via mass spectrometry or cryo-EM in either the TMC-1 (22) or TMC-2 complexes. Likewise, we found no evidence for the presence of PEZO-1, the worm homolog of PIEZO1 and 2, thus demonstrating that, at least in worms, TMC-1 or TMC-2 do not form a stable complex with PEZO-1 (25).

**Fig. 1.**
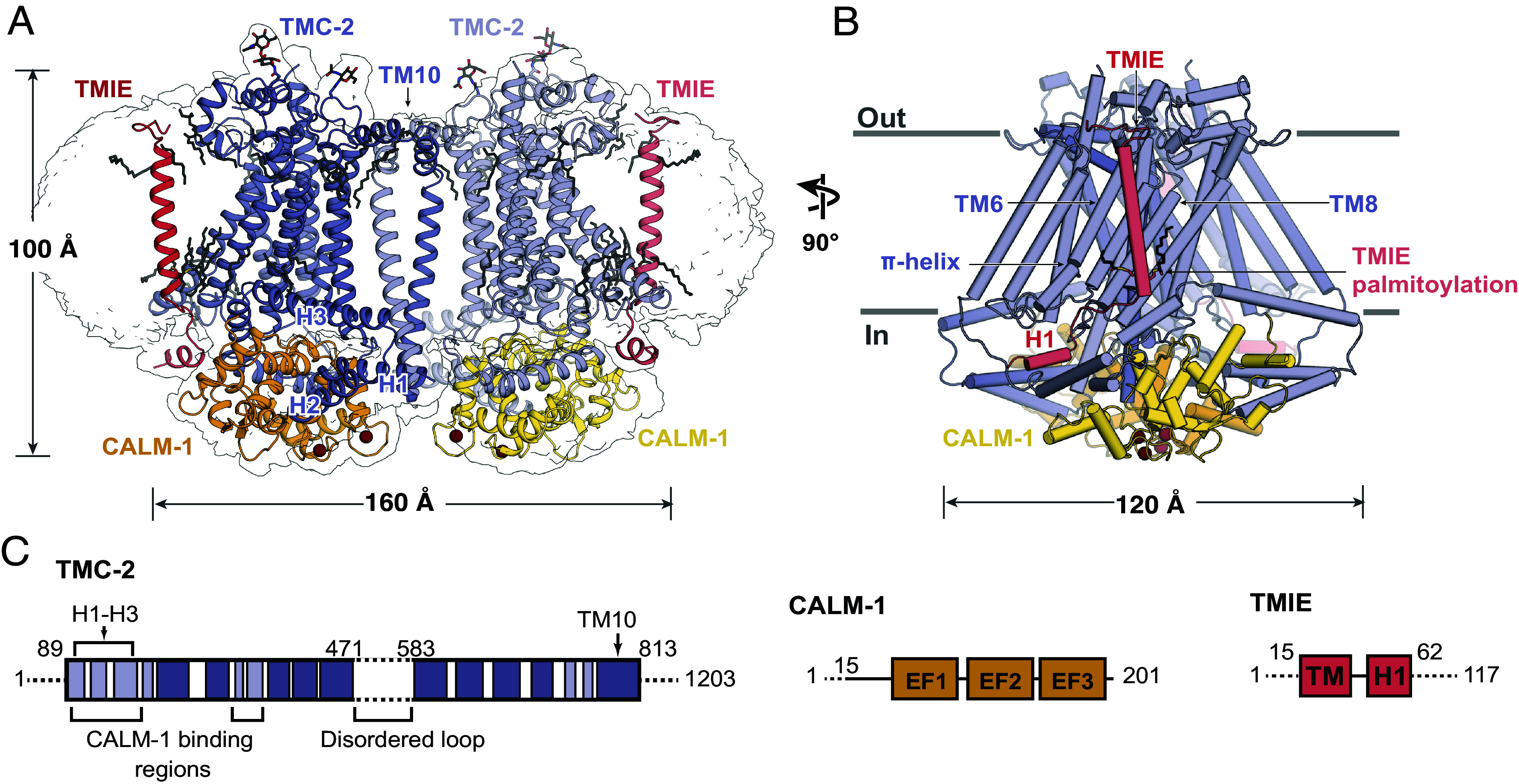
Architecture of the TMC-2 complex. (*A*) Overall architecture of the TMC-2 complex, viewed parallel to the membrane. TMC-2 (dark and light purple), CALM-1 (orange and yellow), and TMIE (dark and light pink) are shown in a cartoon diagram. Lipid-like molecules and *N*-glycans are colored gray and calcium ions are colored red. The map refined without a mask is shown as a transparent envelope. (*B*) A 90° rotated view of the TMC-2 complex, viewed parallel to the membrane. Structural features of interest are indicated. α-helices are shown as cylinders. (*C*) Schematic representation of proteins that form the TMC-2 complex. For TMC-2, helices are shown in light purple, and transmembrane helices are shown in dark purple. Dashed lines indicate regions that were not observed in the cryo-EM structure. H = helix, TM = transmembrane domain, EF1-EF3 = EF-hand domains.

To elucidate the architecture of the native TMC-2 complex, we performed single-particle cryoelectron microscopy (cryo-EM) (*SI Appendix*, Fig. S2 and Table S1). Approximately 100 ng of protein was isolated from *tmc2::mVenus* worms and placed on 2 nm carbon grids that had been glow discharged in the presence of amyl amine. Rounds of 2D classification of the selected particles revealed a high degree of heterogeneity that was not observed in the TMC-1 dataset, despite nearly identical sample and grid preparation methods for TMC-2, including the use of the same detergent as employed in the isolation of TMC-1 (22). The majority of TMC-2 particles were monomeric and exhibited a preferential “top-down” orientation. We also observed an apparent “trans-dimer” conformation that we discarded in 2D classification (*SI Appendix*, Fig. S2*B*). Particles that corresponded to a *cis*-dimeric complex were selected for subsequent refinement and 3D reconstruction, yielding a density map at 3.2 Å resolution, sufficient for model building (Fig. 1 and *SI Appendix*, Fig. S3). Although we attempted to reconstruct the monomeric and trans-dimer conformations of the TMC-2 complex, we were not successful. This may be due to a combination of small protein size, possible conformational heterogeneity, and low particle numbers.

**Fig. 2.**
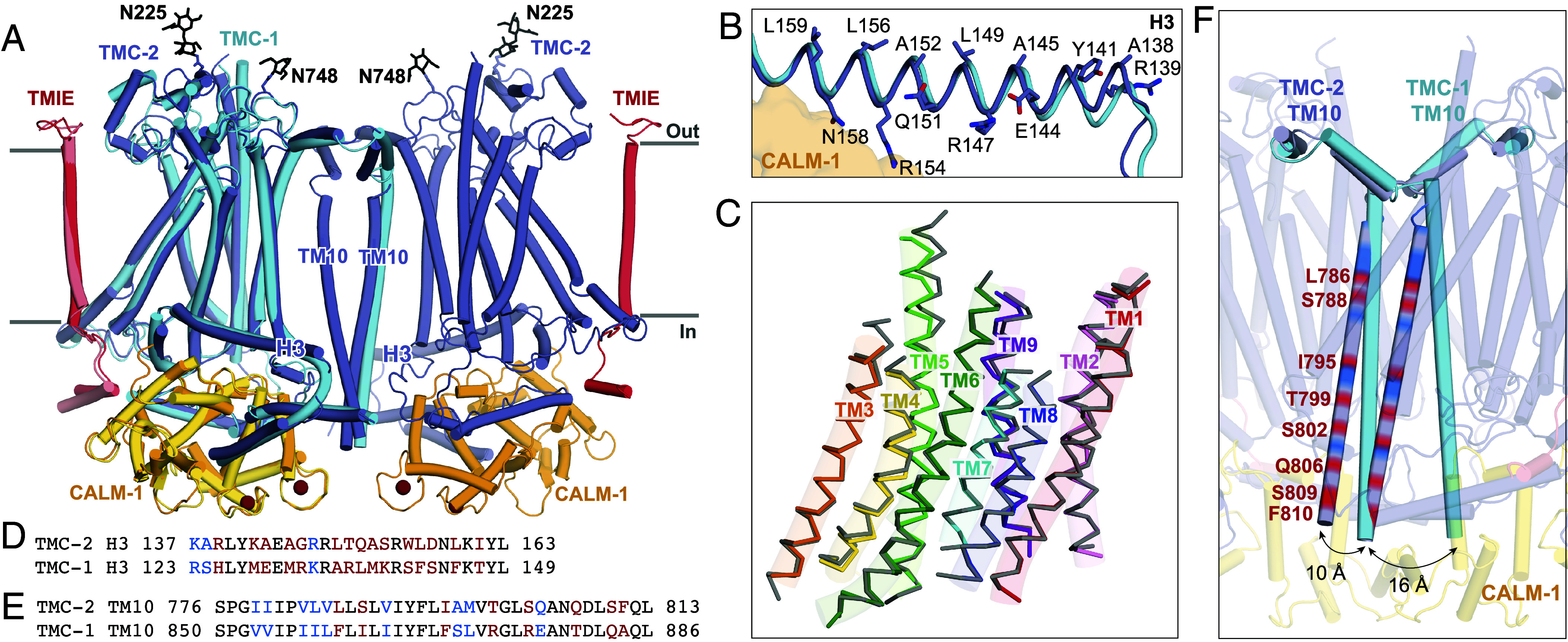
The TMC-1 and TMC-2 complexes have similar architectures, yet adopt distinct conformations at the dimer interface. (*A*) Superposition of backbone atoms of the TMC-2 complex with backbone atoms of one protomer of the TMC-1 complex, shown on the left. TMC-1 is colored cyan and the CALM-1 and TMIE auxiliary subunits from the TMC-1 complex are colored yellow and pink, respectively. The TMC-2 complex is colored as in Fig. 1. *N*-glycan molecules are colored black, and calcium ions are colored dark red. (*B*) TMC-2 helix H3, shown in purple, is superposed with TMC-1 helix H3, shown in cyan. Residues that confer an amphipathic nature upon the TMC-2 H3 helix are shown as sticks. (*C*) The TMC-1 transmembrane helices (TM1-TM9), shown in gray, superposed on the TMC-2 transmembrane helices, shown in different colors, using backbone carbon atoms. (*D*) Sequence alignment of the amino acid residues of the TMC-2 and TMC-1 H3 helices. Residues are colored black for identical, blue for conservatively substituted, or red for nonconservatively substituted. (*E*) Sequence alignment of amino acid residues of the TMC-2 and TMC-1 TM10 regions. Residues are colored as in (*D*). (*F*) Closeup view of the dimer interface of the superimposed complex from panel (*A*), rotated ~45° about an axis perpendicular to the membrane plane, highlighting the different conformations of the TM10 regions in the two complexes. The positions of the nonconserved residues in TM10 of TMC-2, compared to TMC-1 and as defined in panel (*E*), are shown as red bands.

The overall architecture of the TMC-2 complex is similar to TMC-1 (Fig. 2). Although TMC-1 and TMC-2 share only 36% sequence identity, superposition of a single protomer from each structure yields a backbone rmsd of 0.9 Å. As in TMC-1, the TMC-2 complex harbors two copies each of TMC-2, CALM-1, and TMIE (Fig. 1*A*). Each TMC-2 protomer consists of 10 transmembrane helices and the dimer interface is composed of domain-swapped transmembrane helix 10 (TM10). The cytosolic domain includes six helices oriented nearly parallel to the membrane plane, all of which form extensive interactions with CALM-1. The transmembrane domain exhibits the highest local resolution (*SI Appendix*, Fig. S3) and is decorated with an abundance of lipids or lipid-like molecules. Density features consistent with glycosylation are observed at N225, located in the loop between TM1 and TM2, as well as N748, located in the loop between TM9 and TM10. Peptides from both the 125-residue loop between TM5 and TM6 and the approximately 400-residue C-terminus were detected by mass spectrometry, but these regions were not visible in our map (Fig. 1*C*). We suspect that this region is not visible due to heterogeneity, as we observed with TMC-1.

### TMC-1 and TMC-2 Are Structurally Conserved Aside from the Dimer Interface.

Alignment of the TMC-1 and TMC-2 complex structures based on backbone atoms of a single TMC-1 or TMC-2 protomer reveals a high degree of structural conservation in most regions of the complex (Fig. 2*A*). The transmembrane helices, TM1-TM9, exhibit a backbone rmsd of 0.58 Å, and the cytoplasmic domain, residues 89 to 178 and 307 to 366 in TMC-2, exhibits a backbone rmsd of 0.74 Å. Alignment of the auxiliary proteins TMIE and CALM-1 from each complex yields a backbone rmsd of 0.48 Å and 0.46 Å, respectively. The side chain conformations of conserved amino acids in the TMC-1 and TMC-2 transmembrane helices are remarkably similar, although several nonconservative substitutions are apparent in the cryo-EM density map that allow us to easily distinguish between the density maps and their corresponding structures (*SI Appendix*, Fig. S4).

A close comparison of the TMC-1 and TMC-2 complexes, however, reveals several notable differences. On the extracellular side of the complexes, all of the short loops and helices that link transmembrane domains are conserved at a sequence and structural level, aside from the long, unstructured loop that connects TM5 and TM6. This loop, which was not visible in either the TMC-1 or TMC-2 maps, is 75 residues shorter in TMC-2 and only exhibits 14% sequence identity to the TMC-1 loop. Further, while TMC-1 and TMC-2 are both glycosylated at N209 and N225, respectively, we observed a second density feature at a predicted glycosylation site in the TMC-2 map, located on N748 between TM8 and TM9 (Fig. 2*A*). TMC-1 bears an arginine residue at this position (R821) and the surrounding four residues are also different in amino acid sequence (*SI Appendix*, Fig. S5).

The six cytoplasmic helices, all of which interact with the auxiliary protein CALM-1, are structurally conserved between TMC-1 and TMC-2. The helices that share the greatest degree of sequence identity are also those that form the majority of the interactions with CALM-1: H1, H2, H4, and H6. Helix H3, by contrast, only shares 33% sequence identity between TMC-1 and TMC-2 (Fig. 2*D*). Despite the lack of sequence conservation, helix H3 retains the amphipathic nature that was observed in the TMC-1 structure (Fig. 2*B*). Hydrophobic residues on the membrane-proximal side of H3 allow it to interact with the inner leaflet of the membrane bilayer, while polar and charged residues occupy the cytoplasmic side, several of which interact with CALM-1 (R154 and N158). Amphipathic, cytosolic helices that are oriented parallel to the membrane bilayer have been observed in the structures of other mechanically activated channels, such as OSCA1.2 (26) and PIEZO (27), and have been proposed to be a mechanistically important feature of mechanosensitive ion channels (1).

Superposition of the backbone atoms of TM1-TM9 from TMC-1 and TMC-2 reveals nearly identical positions for the transmembrane helices (Fig. 2*C*) and sequence identity among the TMs ranges from 40-86%. However, TM10, which mediates the domain-swapped dimer interfaces, is structurally dissimilar in the TMC-1 and TMC-2 complexes (Fig. 2 *E* and *F* and *SI Appendix*, Fig. S6*A*). Unlike TMC-1, TMC-2 is not C2 symmetric owing to the orientation of the TM10 helices, which are offset by 10-16 Å relative to the TMC-1 TM10 helices (Fig. 2*F*). TM10 exhibits 52% sequence identity between TMC-1 and TMC-2 (Fig. 2*E*), but the side chains of the nonconserved amino acids are directed away from the dimer interface. Dimerization of TMC-2 results in burial of 1,613 A^2^ of solvent-accessible surface area, slightly less than the 1,781 A^2^ of surface area that is buried upon TMC-1 dimer formation, suggesting that fewer contacts exist to stabilize the TMC-2 dimer. Based on secondary structure prediction and AlphaFold2 analysis, the TM10 helices of TMC-1 and TMC-2 traverse the lipid bilayer and extend into the cytosol, forming an elongated coil-like structure that spans approximately 70 Å and is evolutionarily conserved from nematodes to humans. Sequence alignment of TM10 reveals two conserved positively charged amino acids, R873 and R876 in *C. elegans* TMC-1, and K721 and K724 in human TMC1, which are substituted for T800 and S803 in TMC-2. These residues are located proximal to the inner leaflet of the membrane bilayer and may stabilize the TMC-1 dimer through interactions with phospholipids (*SI Appendix*, Fig. S6*B*). The substitution of these positively charged residues for polar residues in TMC-2 may result in increased flexibility of TM10. Further, the ~400 C-terminal residues that follow TM10, which were not visible in the cryo-EM map, only share 22% sequence identity with TMC-1. It is possible that differences in the structure or flexibility of the TMC-1 and TMC-2 C termini may also indirectly impact the conformation of TM10.

### Auxiliary Protein Binding Interfaces and Lipid-Mediated Interactions Are Conserved between TMC-1 and TMC-2.

**Fig. 3.**
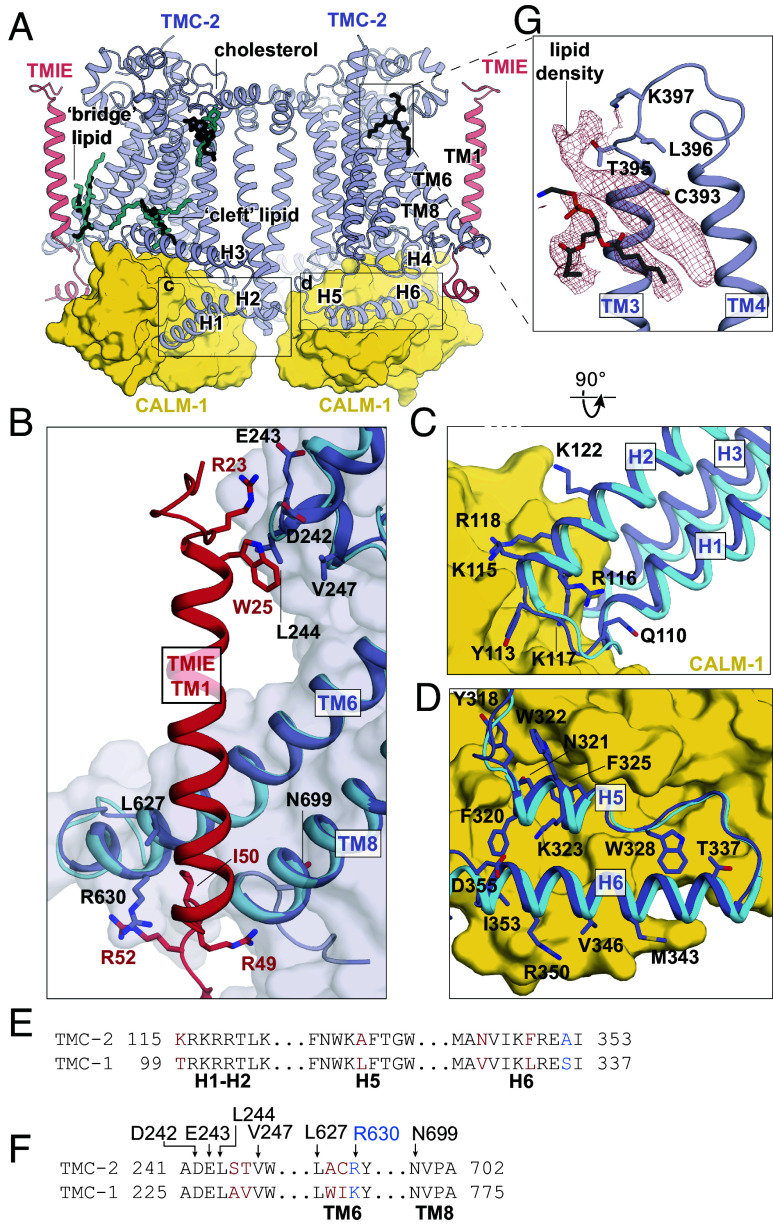
TMC-1 and TMC-2 share a conserved interaction interface with the auxiliary subunits TMIE and CALM-1. (*A*) Lipids with strong density features and similar locations in the TMC-1 and TMC-2 density maps are shown in the context of the TMC-2 complex. TMC-1 lipids are cyan and TMC-2 lipids are black. (*B*) The subunit interface of TMIE in the TMC-1 or TMC-2 complex. TMC-2 is purple, TMC-1 is cyan, and TMIE is red. Interacting residues are shown as sticks. (*C*) The subunit interface of CALM-1 with TMC-1 or TMC-2 helices H1-H2. CALM-1 is colored yellow, and interacting residues are shown as sticks. (*D*) The subunit interface of CALM-1 with TMC-1 or TMC-2 helices H5-H6, colored as in (*C*). (*E*) Sequence alignment of the residues from the cytoplasmic helices of TMC-1 and TMC-2 that form the interaction interface with CALM-1. Residues in black are conserved. Residues in blue are conservatively substituted and residues in red are not conserved. (*F*) Sequence alignment of TMC-2 and TMC-1 residues that interact with TMIE. Residues are colored as in (*E*). (*G*) Lipid-like density features observed in the TMC-2 map that were not observed in the TMC-1 map. The density map is shown in red, and TMC-2 is shown in purple.

Mass spectrometry results indicate that *C. elegans* TMC-2 copurifies with the auxiliary proteins CALM-1 and TMIE (*SI Appendix*, Fig. S1), both of which are predicted components of the vertebrate MT complex (5, 28, 29). In vertebrates, TMIE is known to play an important role in ion channel gating (30), while the specific role of CIB2 and CIB3, the vertebrate homologs of CALM-1, in mechanosensory transduction is less well understood. Mechanotransduction currents are abolished in mice with null mutations in CIB2 and CIB3, resulting in deafness (28, 31, 32). In mice that harbor a TMIE knockout mutation, mechanotransduction currents are reduced or abolished (29, 30, 33). The *C. elegans* TMC-1 and TMC-2 complexes share nearly identical binding interfaces for CALM-1 and TMIE, emphasizing the importance of these auxiliary protein interactions for MT complex function (Fig. 3 *A–E*). CALM-1 interacts with all six TMC-2 cytoplasmic helices through an extensive network of hydrogen bonds, electrostatic interactions, and hydrophobic contacts. The short loop that connects TMC-2 H1 and H2 harbors an abundance of basic residues that interact with an acidic patch on CALM-1 (Fig. 3*C*) and TMC-2 H4-H6 are bound to CALM-1 through a mixture of polar and nonpolar interactions (Fig. 3*D*). Nearly all of the TMC-1 and TMC-2 residues that mediate interaction with CALM-1 are conserved (Fig. 3*E*), highlighting the high degree of conservation in the CALM-1/TMC binding interface. Likewise, the regions of TMC-1 and TMC-2 that mediate interaction with TMIE are conserved, both in structure and in sequence (Fig. 3 *B* and *F*). Arginine residues in the cytoplasmic TMIE “elbow” form hydrogen bonds with backbone atoms of TMC-2 TM6 and TM8, and TMIE R23 and W25 residues on the extracellular side interact with the loop between TMC-2, TM1 and TM2. TMIE also makes lipid-mediated hydrophobic contacts with the TMC-2 transmembrane domains through phospholipid molecules and palmitoylation of two TMIE cysteine residues. These lipid features and contact sites are conserved and are discussed in the following sections.

Multiple ordered lipids and lipid-like molecules decorate the TMC-1 and TMC-2 transmembrane domains (Fig. 1*A*). Remarkably, many of the lipids with the strongest density features are found in the same locations in both structures (Fig. 3*A*), suggesting that they play a role in complex stability or ion channel function, or both. A phospholipid occupies the cleft between TMC-2 H3 and TM1 (“cleft” lipid), and another resides in the cavity between the TMIE transmembrane helix and TMC-2 TM6 and TM8 (“bridge” lipid). The location of the bridge lipid between the TMIE and TMC-1/2 transmembrane helices is of special interest because it makes hydrophobic contacts between structural elements that are predicted to be involved in ion channel gating. An additional density feature that is consistent with a cholesterol molecule resides proximal to the extracellular side of TM2 in both structures. Interestingly, we observed a prominent lipid-like density in the TMC-2 map that is not present in TMC-1 (Fig. 3*G*). The lipid-like feature resides in the extracellular leaflet, occupying a crevice between TM3 and TM4. Further analysis, and possibly higher resolution reconstructions, will be required to determine the molecular composition of this lipid-like density feature.

### The Putative Pore of TMC-2 Is Distinct from TMC-1.

**Fig. 4.**
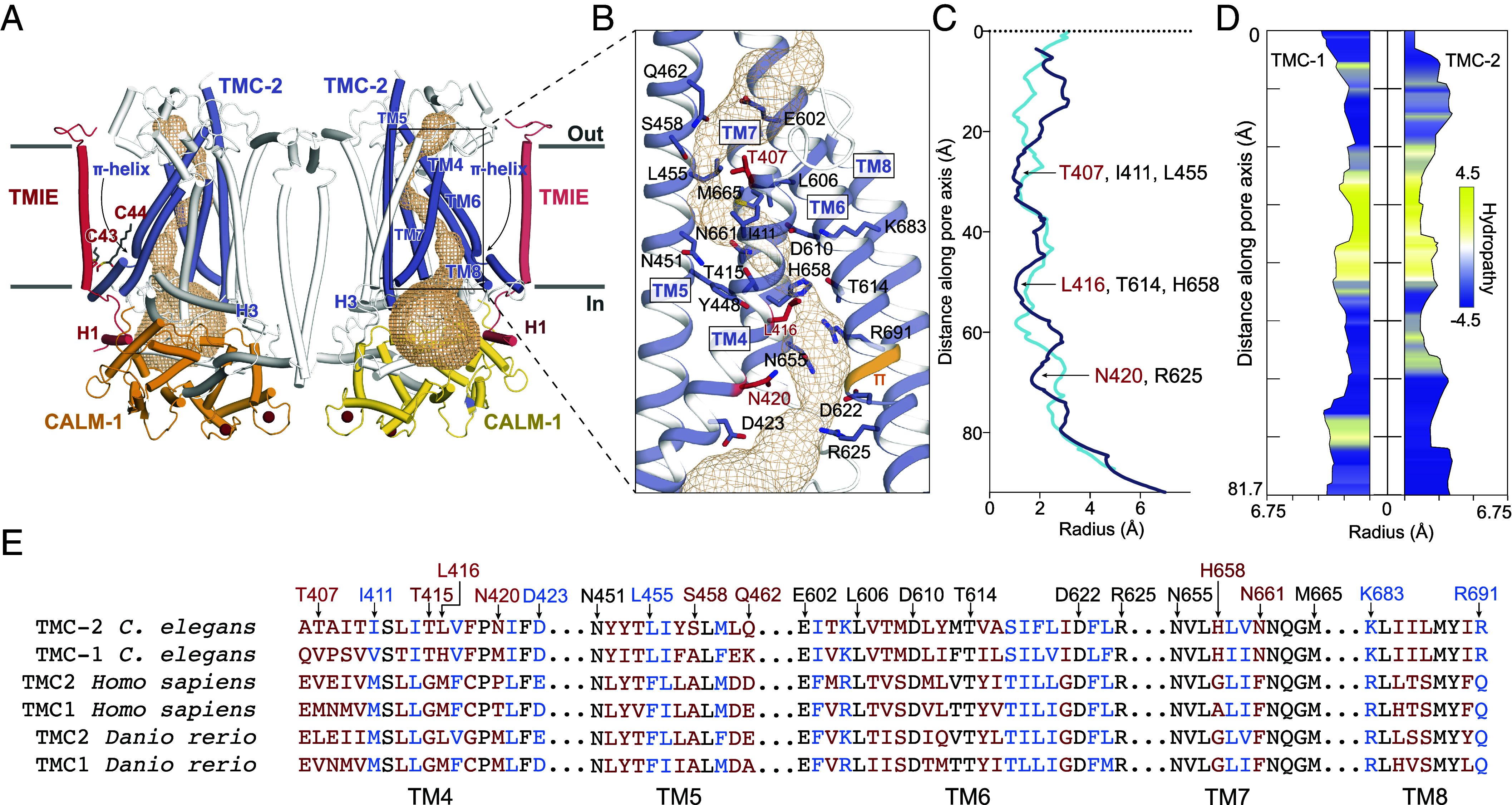
The putative ion conduction pore of TMC-2. (*A*) The putative location of the pore is shown as gold mesh. The pore-lining helices, TM4-8, are shown in purple, and the remaining TM helices are shown in white. (*B*) An expanded view of the possible ion permeation pathway, showing pore-lining residues as sticks. Residues that are not conserved between TMC-1 and TMC-2 are shown in red. A π-helical turn within TM6 of TMC-2 is shown in orange. (*C*) Radii of the TMC-1 and TMC-2 pores plotted along the pore axis, calculated by Mole 2.5. TMC-1 is shown in blue, and TMC-2 is shown in purple. (*D*) Hydropathy plot of the TMC-1 pore (*Left*) and TMC-2 pore (*Right*) calculated by Mole 2.5. Regions of the pore with high hydrophobicity values are colored yellow, and regions with high hydrophilicity values are colored blue. The size of the pore is plotted along the pore axis. (*E*) Multiple sequence alignment of selected residues from the putative TMC-2 and TMC-1 pore-lining helices of different species, with the residues colored as in panel (*B*).

The putative ion conduction pathway of TMC-2 follows the same trajectory as the conduction pathways in TMC-1 and the evolutionarily related proteins TMEM16A (34, 35) and OSCA1.2 (26, 36) (Fig. 4*A*). Like the structure of TMC-1, the ion conduction pore is lined by TM4-8. The pore appears closed and is blocked by three sites of constriction (Fig. 4 *B* and *C*). The extracellular pore entrance, which is lined by a mixture of polar and nonpolar residues, is followed by a narrow “neck” region that is dominated by hydrophobic residues (Fig. 4*D*). The remainder of the pore, toward the cytoplasm, is lined by polar and charged residues (Fig. 4 *B* and *D*).

The ion selectivity and permeation properties of the *C. elegans* TMC-1 and TMC-2 ion channels are not known, in large part due to an inability to express functional channels in a heterologous system. Studies of vertebrate TMC1 and TMC2 channels in hair cells have demonstrated that, while both proteins form cation-selective ion channels (37) with a high permeability for calcium (38), their electrophysiological properties differ in several ways. TMC2-expressing hair cells differ in their single-channel conductance, calcium permeability, adaptation rates, and permeability for small molecules relative to TMC1-expressing cells (2, 9, 39), suggesting that the electrophysiological properties of *C. elegans* TMC-1 and TMC-2 may differ as well. Analogous to *C. elegans* TMC-1, the composition of *C. elegans* TMC-2 pore-lining residues is not consistent with a cation-selective channel, which usually harbors a predominance of acidic residues. While four highly conserved acidic residues line the TMC-2 pore, there are also four basic residues scattered throughout the putative conduction pathway, two of which are conserved across multiple species (Fig. 4 *B* and *E*). It is unclear whether these residues line the pore in its open conformation.

The majority of the pore-lining residues are conserved between TMC-1 and TMC-2, nevertheless. Intriguingly, TM4 contains three of the five residues that are not conserved. These amino acid differences all involve substitution of a polar or charged residue for a nonpolar residue. Mutagenesis studies of vertebrate TMC1 in hair cells have demonstrated that TM4 and TM6 are important for mechanical gating of TMC1 (40), suggesting that the aforementioned nonconservative substitutions in TM4 may alter the mechanical activation of *C. elegans* TMC-2 relative to TMC-1. Further, in contrast to the TMC-1 complex, we did not observe spherical densities corresponding to putative Ca^2+^ ions near acidic residues D610 and D622 (D683 and D695 in TMC-1). These two aspartate residues are conserved in human TMC1 and TMC2, suggesting that they are important for ion coordination (Fig. 4*E*). It is possible that the putative bound cations were observed in the TMC-1 complex, but not the TMC-2 complex, due to differences in pore-lining residues, which may change the permeability properties of TMC-2 relative to TMC-1.

### Palmitoylation of TMIE Suggests a Mechanism for TMC-1/2 Gating.

**Fig. 5.**
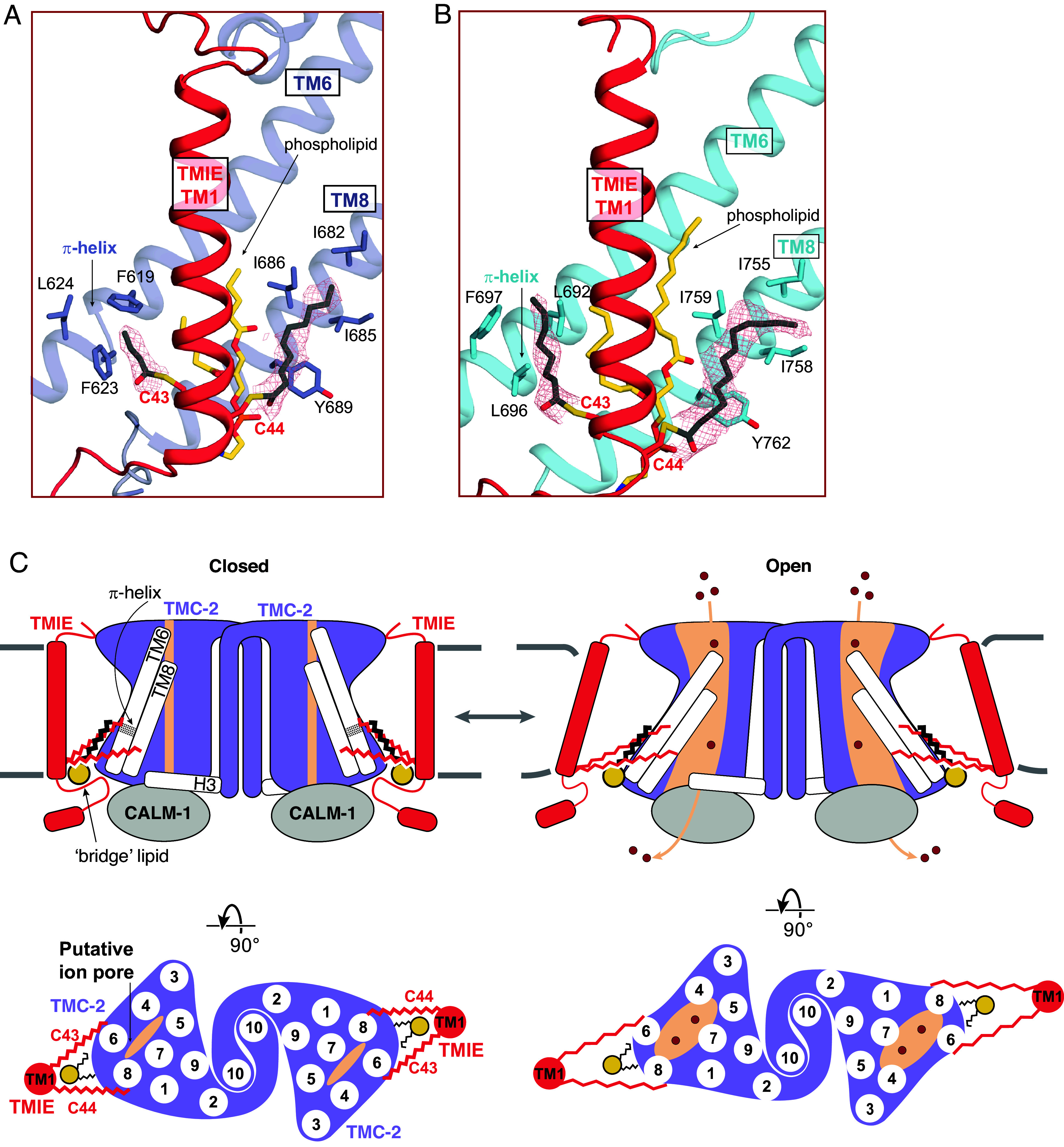
Lipid-mediated interactions between TMIE and TMC-1/2. (*A*) Palmitoylation of TMIE C43 and C44 is observed in the TMC-2 complex. Interacting residues are shown as sticks, and a phospholipid is shown in yellow. Cryo-EM density is shown as red mesh at a contour level of 5.68 rmsd. (*B*) Palmitoylation of TMIE C43 and C44 is also observed in the TMC-1 complex. TMC-1 is shown in cyan, and other coloring is depicted as in (*A*). The density map contour level is 4.62 rmsd. (*C*) Schematic illustrating how lipid-mediated interactions between TMIE and the TMC-1/2 transmembrane helices may translate forces to ion channel gating. Lipid interactions may stabilize the closed conformation of the channel (*Left*). Disruption of the membrane due to mechanical forces could alter these lipid interactions, leading to ion channel opening (*Right*). Phospholipid head groups are colored yellow.

Studies in vertebrate hair cells have demonstrated that TMIE alters the gating properties, unitary conductance, and ion selectivity of the vertebrate MT complex (30). However, currents with reduced amplitude can still be observed in TMIE knockout mice, demonstrating that TMIE is not essential for mechanotransduction (33). The structure of the *C. elegans* TMC-1 complex revealed that TMIE C44 is palmitoylated and the acyl chain extends toward TMC-1 pore-forming transmembrane helix TM8, potentially linking TMIE to the ion conduction pathway. We observed a second TMIE palmitoylation site on C43 in the TMC-2 complex structure that may have additional implications for gating of TMC ion channels (Fig. 5*A*). Prompted by the palmitoylation of TMIE in the TMC-2 complex, we reinspected the cryo-EM density maps of the TMC-1 complex and found evidence that TMIE C43 is also palmitoylated (Fig. 5*B*). The palmitoyl groups of C43 and C44 are oriented almost identically in both the TMC-1 and TMC-2 complexes; the acyl chain of C43 extends in the direction of TM6 and the acyl chain of C44 makes hydrophobic contacts with nonpolar residues in TM8 (Fig. 5). Interestingly, the palmitoyl groups of TMIE exhibit a conformation in which their acyl groups embrace the conserved bridge lipid. While the density for a palmitoyl group on C43 in both the TMC-1 and TMC-2 reconstructions is weaker than the corresponding density associated with C44, we were nevertheless able to confidently fit a palmitoyl moiety to the density. Because the density for the palmitoyl group of C44 is stronger, we speculate that the two palmitoyl groups differ in their conformational mobility. How these differences are related to the function of the complex remains to be determined.

Notably, the C43 palmitoyl group contacts two phenylalanine residues, F619 and F623, that are positioned on a π-helical turn of TMC-2 TM6. The π-helical turn is conserved in the TMC-1 structure, but the phenylalanine residues are replaced by leucine residues, L692 and L696 (Fig. 5*B*). TMC-1 F697 further bolsters the hydrophobic interaction with the palmitoyl group. A leucine or phenylalanine residue is conserved in all three positions across multiple species (Fig. 4*E*), indicating that these residues may be important for the indirect interaction between TMIE and TMC-1/2. Palmitoyl groups are known to mediate protein–protein interactions in diverse cellular systems (41–45). As TMC1/2 TM6 plays a role in vertebrate ion channel gating (40), the arrangement of TMIE C43 palmitoylation with respect to the TM6 π-helical turn suggests that could be important for gating of the ion channel.

## Discussion

The isolation and elucidation of the molecular structure of the native *C. elegans* TMC-2 complex, while similar to the TMC-1 complex, reveals several key features that may render it functionally distinct from TMC-1. First, the composition of the TMC-1 and TMC-2 complexes is not identical; the auxiliary subunit ARRD-6 does not copurify with the TMC-2 complex, which could be due to differences in expression patterns between TMC-1 and TMC-2. Second, TMC-1 and TMC-2 differ in the structure of the dimer interface, an observation that is intriguing due to the high degree of structural conservation between TMC-1 and TMC-2 in all other regions of the proteins. Third, we observed key differences in the composition of pore-lining amino acids, 75% of which are localized to TM4, a pore-lining helix that is important for gating in vertebrate TMC1.

Mass spectrometry and cryo-EM experiments of TMC-1 and TMC-2 provide further insight into the subunit composition of these complexes. Our results indicate that TMC-1 and TMC-2 do not form heterodimers in *C. elegans*, despite their conserved complex architecture and common localization to worm body wall and vulval muscles. We note that there is a size difference between the expected molecular weight of the TMC-2 complex and the size of the complex estimated by FSEC. We speculate that the discrepancy between the predicted and observed molecular weight may be due to rapid unbinding of labile auxiliary subunits, such as the worm homologs of MT subunits protocadherin-15 (PCDH15, cdh-5 in worms) and lipoma HMGIC fusion partner-like 5 (LHFPL5, lhfp-4 in worms), or perhaps more likely, due to the presence of lipids and a large detergent micelle. Although worms express orthologs of PCDH15 and LHFPL5, we did not observe copurification of these subunits with worm TMC-1 and TMC-2. In vertebrates, PCDH15 forms part of the extracellular tip-link that connects adjacent stereocilia in a hair cell and is thought to convey force to the MT complex, thus playing an essential role in mechanosensation (46, 47). LHFPL5 forms a complex with PCDH15 in vitro (48–50) and null mutations in mouse LHFPL5 reduce MT currents (49, 50). While it is possible that the lack of worm TMC-1/2 copurification with PCDH15 or LHFPL5 is due to complex dissociation during purification, it is also conceivable that worms rely on a different mechanism of force-sensing that does not involve an extracellular tether.

Further, we observed an unexpectedly high proportion of monomeric TMC-2 complexes in our cryo-EM studies, which may be related to heterogeneity of the dimer interface. It is worth noting, however, that recent structural analyses have revealed unexpected oligomeric states of two other ion channels: transient receptor potential channel 3 (TRPV3) and TMEM63C. All known TRP channels are tetramers, but TRPV3 presents as a pentamer (51). Cryo-EM studies of TMEM63C, a mammalian homolog of the dimeric OSCA ion channel, demonstrate that it is primarily a monomer in solution (52). The oligomeric state of these ion channels in their native environment is unknown.

Finally, the presence of a π-helical turn in the likely pore-lining TMC-1/2 TM6 helix, and its interaction with the TMIE C43 palmitoyl group, may have implications for a gating mechanism of TMC-1/2 (Fig. 5*C*). The π-helix structural motif is present in ~15% of proteins and is typically associated with a specific protein function (53, 54), including ion channel gating (55, 56). We hypothesize that the π-helical turn plays a role in mechanical gating of TMC-1/2, possibly by undergoing a π- to α-helix transition. In support of this speculation, the ion channels OSCA1.2 and TMEM16, both of which are evolutionarily related to TMC proteins (13, 57–59), also harbor π-helical turns in their pore-forming helices. The mechanosensitive ion channel OSCA1.2 bears two π-helical turns in TM5 and TM6a (26), and the mouse Ca^2+^-activated chloride channel TMEM16A and the fungus lipid scramblase TMEM16 each harbor a π-helical turn in TM6 (34, 56). The gating mechanism of TMEM16A relies on an α-to-π transition, wherein Ca^2+^ binding stabilizes the open conformation of the channel via Ca^2+^ interaction with the π-helical turn (56). It is interesting to speculate that the TMC complexes have a similar gating mechanism. The interaction between the TMIE C43 palmitoyl group and hydrophobic residues on the TMC π-helical turn may stabilize the π-helix, thereby stabilizing the closed conformation of the channel. Deformation of the membrane through mechanical forces could disrupt the interaction between the palmitoyl group and TM6, enabling a π-to-α transition that gates the ion channel.

Together, the conserved protein–lipid interactions between TMIE and TMC-1/2 allow us to build upon our proposed model of TMC complex mechanical activation (Fig. 5*C*). The TMIE C43 and C44 palmitoyl groups interact with the pore-forming helices TM6 and TM8, as well as with the conserved bridge phospholipid, which contacts both the TMC and TMIE transmembrane domains. These extensive lipid-mediated hydrophobic contacts may functionally link TMIE and TMC-1/2. We hypothesize that when force is applied to TMIE, either through membrane stretch or the action of an auxiliary subunit, those forces are translated to the TMC pore through lipid-mediated interactions, leading to channel opening and ion permeation.

## Materials and Methods

### Transgenic Worm Design.

The strain PHX4776 *tmc-2(syb2106syb4776)* was generated by SunyBiotech using CRISPR/Cas9 genome editing and is referred to as the *tmc2::mVenus* line. A nucleic acid sequence encoding a mVenus-3xFLAG tag was inserted prior to the stop codon of the endogenous *tmc-2* gene (Wormbase: B0416.1a). The genotype was confirmed using PCR and primers ER14-seq-s (TGAACCGCATCGAGCTGA) and ER14-seq-a (CAAAAGTGGCCAATTTCGGG), which bind 104 bp upstream and 360 bp downstream from the insertion site, respectively, to amplify the region of interest. To enable elution of the engineered TMC-2 complex from affinity chromatography resin, a nucleic acid sequence coding for a PreScission protease (3C) cleavage site was placed between elements encoding the mVenus fluorophore and the 3xFLAG tag.

### Spectral Confocal Imaging.

Adult worms were immobilized in M9 buffer (22 mM KH_2_PO_4_, 42 mM Na_2_HPO_4_, 86 mM NaCl, and 1 mM MgCl_2_) containing 30 mM sodium azide and placed on slides that were prepared with ~4-mm agar pads. Spectral images were acquired on a Zeiss 34-channel LSM 880 Fast Airyscan inverted microscope with a 40× 1.2 NA water-immersion objective lens. Linear unmixing was employed to distinguish between the mVenus signal and autofluorescence. The autofluorescence signal was subtracted from each image. The 3D z-stack information is presented in 2D after performing a maximum intensity projection.

### Large-Scale *C. elegans* Culture.

Large-scale worm growth and optimization of TMC-2 protein isolation conditions were performed as described in Clark et al. (23). Briefly, nematode growth medium (NGM) agar plates were prepared and spread with *Escherichia coli* strain HB101, allowing the bacterial lawn to grow overnight at 37 °C. Worms were transferred to the NGM plates and grown for 3 to 4 d at 20 °C until HB101 cells were depleted. Worms on the plates were transferred to a liquid medium in 2 L baffled flasks, supplemented with HB101 (~15 g per 500 mL medium) and streptomycin (50 μg/mL), and worms were grown at 20 °C with vigorous shaking (150 rpm) for 70 to 72 h. To harvest worms, the liquid culture flasks were placed on ice for 1 h to allow the worms to settle. The medium was removed, and the worm slurry was collected in a tube, washed twice with 50 mL of ice-cold M9 buffer by successive centrifugation (800 × g for 1 min), and resuspended. Worms were “cleaned” by sucrose density centrifugation at 1,500 × g for 5 min after bringing the volume of worm slurry up to 25 mL with M9 buffer and adding 25 mL of ice-cold 60% (w/v) sucrose. The worm layer on top was recovered and placed in a new tube and then washed once with 50 mL of ice-cold M9 buffer and once with 50 mL of lysis buffer (50 mM Tris-Cl, pH 8.0, 50 mM NaCl, 5 mM EDTA, and protease inhibitors that included 0.8 µM aprotinin, 2 µg/mL leupeptin, and 2 µM pepstatin). The volume of the worm pellet was measured, and the same volume of lysis buffer was added to the tube and worm balls were made by dripping the slurry into liquid nitrogen. The worm balls were stored at −80 °C until further use.

### Isolation of the Native TMC-2 Complex.

The experimental procedure for isolation of the TMC-2 complex is similar to the isolation procedure for the TMC-1 complex (22) but is nonetheless described in detail below. Approximately 50 g of frozen worm balls was disrupted using a ball mill (MM400, Retsch) where the grinding jar and ball were precooled in liquid nitrogen. Disrupted worm powder was solubilized at 4 °C for 2 h in a buffer containing 50 mM Tris-Cl (pH 8.0), 50 mM NaCl, 5 mM EDTA, 2% (w/v) glyco-diosgenin (GDN), and protease inhibitors (0.8 µM aprotinin, 2 µg/mL leupeptin, and 2 µM pepstatin). The supernatant was clarified by centrifugation at 40,000 rpm (186,000 × g) for 50 min, followed by filtration with a 0.45-µm filter. The clarified supernatant was applied to anti-FLAG M2 magnetic affinity resin and incubated overnight on a rotator at 4 °C. The resin was washed 5 times with a buffer containing 20 mM Tris-Cl (pH 8.0), 150 mM NaCl, and 0.02% (w/v) GDN, using a volume of buffer that was 200-fold the volume of the resin. The TMC-2 complex was eluted by incubating the resin with 40 μg of 3C protease at 4 °C for 2 h, on a rotator. Subsequently, the solution was supplemented with 1 mM CaCl_2_, final concentration, and the concentrate was loaded onto a size-exclusion chromatography (SEC) column (Superose 6 Increase 10/30 GL, GE Healthcare) connected to a Shimadzu high-pressure liquid chromatography instrument. The column was equilibrated in a buffer composed of 20 mM Tris-Cl (pH 8.0), 150 mM NaCl, 0.02% (w/v) GDN, and 1 mM CaCl_2_, and the protein elution was monitored by tryptophan fluorescence (excitation= 280 nm; emission = 350 nm). The peak fractions from the putative dimeric TMC-2 complex were collected by hand, pooled, and concentrated for cryo-EM grid preparation. Approximately 100 ng of TMC-2 was isolated from 50 g of worm balls or about 3.8 × 10^7^ worms. The amount of protein was determined via mVenus fluorescence based on a standard plot. The isolated native TMC-2 sample was analyzed by SDS-PAGE (sodium dodecyl sulfate–polyacrylamide gel electrophoresis), and the protein bands were visualized by silver staining. For mass spectrometry analysis, the putative dimeric TMC-2 complex peak was pooled and concentrated to a volume of 200 µL.

### Mass Spectrometry.

The SEC-purified TMC-2 complex sample was dried and then dissolved in a solution consisting of 5% sodium dodecyl sulfate, 8 M urea, and 100 mM glycine (pH 7.55). The sample was then reduced with [tris(2-carboxyethyl)phosphine (TCEP)] at 37 °C for 15 min and alkylated with methyl methanethiosulfonate for 15 min at room temperature, followed by addition of acidified 90% methanol and 100 mM triethylammonium bicarbonate buffer (TEAB; pH 7.55). The sample was then digested in an S-trap micro column briefly with 2 µg of a Tryp/LysC protease mixture, followed by a wash and 2-h digestion at 47 °C with trypsin. The peptides were eluted with a solution consisting of 50 mM TEAB, 50% acetonitrile and0.2% formic acid, and were then pooled and dried. Each sample was dissolved in 20 µL of 5% formic acid and injected into the Thermo Fisher QExactive HF mass spectrometer. Protein digests were separated using a Dionex RSLC UHPLC system, then delivered to a QExactive HF (Thermo Fisher) via electrospray ionization using a Nano Flex Ion Spray Source (Thermo Fisher) that is fitted with a 20-μm stainless steel nano-bore emitter spray tip and a 2.6 kV source voltage. Xcalibur version 4.0 was used to control the system. Samples were applied at 10 µL/min to a Symmetry C18 trap cartridge (Waters) for 10 min, then switched onto a 75 µm × 250 mm NanoAcquity BEH 130 C18 column with 1.7 µm particles (Waters) using mobile phases water (A) and acetonitrile (B) containing 0.1% formic acid and a 7.5 to 30% acetonitrile gradient over 60 min with a 300 nL/min flow rate. Survey mass spectra were acquired over m/z 375 to 1,400 at 120,000 resolution (m/z 200), and data-dependent acquisition selected the top 10 most abundant precursor ions for tandem mass spectrometry by higher energy collisional dissociation using an isolation width of 1.2 m/z, normalized collision energy of 30, and a resolution of 30,000. Dynamic exclusion was set to auto, charge state for MS/MS +2 to +7, maximum ion time 100 ms, minimum AGC target of 3 × 10^6^ in MS1 mode, and 5 × 10^3^ in MS2 mode. Data analysis was performed using Comet (v. 2016.01, rev. 3) (60) against a January 2022 version of canonical FASTA protein database containing *C. elegans* uniprot sequences and concatenated sequence-reversed entries to estimate error thresholds and 179 common contaminant sequences and their reversed forms. Comet searches for all samples were performed with trypsin enzyme specificity. Monoisotopic parent ion mass tolerance was set to 1.25 Da and monoisotopic fragment ion mass tolerance was set to 1.0005 Da. A static modification of +45.9877 Da was added to all cysteine residues and a variable modification of +15.9949 Da on methionine residues. A linear discriminant transformation was used to improve the identification sensitivity from the Comet analysis (61, 62). Separate histograms were created for matches to forward sequences and for matches to reversed sequences for all peptides of seven amino acids or longer. The score histograms of reversed matches were used to estimate peptide false discovery rates (FDRs) and set score thresholds for each peptide class. The overall protein FDR was 1.2%.

### Cryo-EM Sample Preparation.

A volume of 3.5 μL of the concentrated TMC-2 complex was applied to a Quantifoil grid (R2/1 300 gold mesh, covered by 2-nm continuous carbon film), which was glow-discharged at 15 mA for 30 s in the presence of amylamine. The grids were blotted and flash frozen using a Vitrobot mark IV for 2.5 s with 0 blot force after 30 s wait time under 100% humidity at 10 °C. The grids were plunge-frozen into liquid ethane that was cooled by liquid nitrogen.

### Data Acquisition.

The native TMC-2 complex dataset was collected on a 300 keV FEI Titan Krios microscope equipped with a K3 detector. The micrographs were acquired in super-resolution mode (0.4155 Å/pixel) with a magnification of 105 k× corresponding to a physical pixel size of 0.831 Å/pixel. Images were collected by a 3 × 3 multihole per stage shift and a six multishot per hole method using Serial EM, with a defocus range of −1.5 to −2.5 µm. Each movie stack was exposed for 5.19 s and consisted of 50 frames per movie, with a total dose of 50 e^−^/Å^2^. A total of 23,364 movies were collected.

### Image Processing.

Beam-induced motion was corrected by patch motion correction with an output Fourier cropping factor of 1/2 (0.831 Å/pixel). Contrast transfer function (CTF) parameters were estimated by patch CTF estimation in CryoSparc v3.3.1 (63). A total of 23,324 movies were selected by manual curation, and the particles were picked using blob-picker with minimum and maximum particle diameters of 140 Å and 200 Å, respectively (*SI Appendix*, Fig. S2*A*). Initially, 4.7 million particles were picked and extracted with a box size of 400 pixels and binned 4× (3.324 Å/pixel). 2D classification of all 4.7 million particles revealed that the TMC-2 complex exists in at least two assembly states, a “cis-dimer” and a “trans-dimer,” as well as in two stoichiometric states, monomer and dimer (*SI Appendix*, Fig. S2*B*). As an initial step to remove TMC-2 complexes that were in the trans-dimer or monomeric states, in addition to junk particles and empty micelles, five rounds of 2D classification were performed, resulting in 1.3 million particles in total. Ab initio reconstruction of the TMC-2 complex from the cleaned particle stack was unsuccessful, likely due to conformational heterogeneity. We expected the overall architecture of the TMC-2 complex to be similar to that of the TMC-1 complex for three reasons: 1) the 2D class averages of TMC-2 and TMC-1 are similar [*SI Appendix*, Fig. S3*B* and reference (22)]; 2) TMC-1 and TMC-2 share 59% sequence identity in their transmembrane domains; and 3) mass spectrometry analysis of the TMC-2 complex suggests that TMC-2 interacts with CALM-1 and TMIE, the same auxiliary proteins present in the TMC-1 complex. As starting models for heterogeneous refinement, we therefore used the “expanded” form of the TMC-1 complex (PDB 7USW) at 3.0 Å resolution and the TMC-1 complex that had been low pass filtered to 10 Å and 20 Å. The full particle stack consisting of 1.3 million particles from 2D classification was subjected to heterogeneous refinement with C1 symmetry using these three starting models in six classes total. A second round of heterogeneous refinement was then performed with the two good classes, which consisted of 623 k particles, to further clean the particle stack. The best class, consisting of 320 k particles, was selected for a final round of heterogeneous refinement, yielding 213 k particles that were re-extracted from unbinned images. To attain higher resolution and improve map quality, nonuniform refinement, including defocus and global CTF refinement, was performed in Cryosparc v3.3.1, resulting in a map with 3.0 Å resolution. One protomer of this map was clearly lower resolution than the other, likely due to conformational heterogeneity of the dimer interface. To further improve the resolution of one protomer, local refinement was performed with a mask covering one half of the complex (one subunit each of TMC-2, CALM-1, and TMIE). This resulted in the “single protomer map” with a resolution of 2.93 Å.

The “protomer” map lacked adequate resolution of TM10 for model building. To attain higher resolution in this region, the re-extracted particles were first symmetry expanded with C2 symmetry. The particles were then subjected to 3D classification without alignment using a mask centered on TM10 with 6 Å target resolution, followed by local refinement. The resulting “dimer interface” map was used for model building of TMC-2 TM10.

### Structure Determination and Model Building.

The protomer map was used for building the entirety of the TMC-2 complex structure aside from TM10 (*SI Appendix*, Fig. S2*A*). The transmembrane helices of TMC-2 (TM1-9, excluding TM10), predicted by Alphafold2 (64) as a template, were fit into the map with rigid body fitting and de novo model building in Coot (65) and Isolde (66). The cytoplasmic and extracellular regions of TMC-2 were built with a combination of de novo model building and rigid body fitting of the TMC-1 structure (PDB 7USW), followed by appropriate modification of the amino acid sequence. The possible ion permeation pore of the channel was determined by MOLE 2.5 (67). Carbohydrate groups were modeled into protruding densities of N225 and N748 on TMC-2 at predicted *N*-linked glycosylation sites. To build the structures of the auxiliary subunits CALM-1 and TMIE, the subunit structures from the TMC-1 complex were fit into the map with rigid body fitting in UCSF Chimera. The TMIE and CALM-1 subunits were then manually adjusted in Coot, followed by real-space refinement in Phenix.

As the protomer map only includes density for one half of the TMC-2 complex, it was necessary to duplicate the map to build the second protomer. The map was rotated 180 degrees and rigid body fit into the “dimer interface” map in UCSF Chimera (68) to produce a map with two copies of each subunit. The model of the TMC-2 complex was then rigid body fit into the map in UCSF Chimera, followed by manual adjustment in Coot. The TM10 helices were built de novo in Coot using the dimer interface map (*SI Appendix*, Fig. S2*A*). The model was refined against the sharpened protomer map by real-space refinement in Phenix.

The following regions of TMC-2 were not modeled into the map because of weak or absent densities: The N-terminal region of TMC-2 (M1 to A88), the predicted loop region between TM5 and TM6 (A472 to T582), and the C-terminal region (M814 to D1203). The side chains with weak density located in the TM10 helices were modeled as alanine residues. The N-terminal region of CALM-1, residues M1-F14, and the N- and C-terminal residues of TMIE, residues M1-L14 and K63-V117, were not modeled due to a lack of density. The N-terminal ~14 residues of TMIE may function as a signal peptide, as proposed in Jeong et al. (22). Peptides corresponding to the N terminus of TMIE were not observed in mass spectrometry data of the TMC-1 complex (22) or of the TMC-2 complex, and electron density for the N-terminal 17 amino acids was not observed in the TMC-1 map. Nevertheless, it is unclear exactly which residues may comprise the signal peptide.

The palmitoylation of TMIE C43 in the TMC-1 complex was built using de novo model building in Coot, based on the cryo-EM volume data previously deposited to the Electron Microscopy Data Bank (22). Real space refinement and regularization were performed in Coot.

## Supplementary Material

Appendix 01 (PDF)

## Data Availability

The volumes for the cryo-EM data have been deposited in the Electron Microscopy Data Bank under accession codes EMD-41356 (69) (“protomer” map) and EMD-41432 (70) (“dimer interface” map). The coordinates have been deposited in the Protein Data Bank under accession code 8TKP (71). The TMC-1 complex coordinates (7USW, 7USX, and 7USY) have been revised to include the palmitoylation of TMIE C43.
