# Supplementary material for: The structure of the Caenorhabditis elegans TMC-2 complex suggests roles of lipid-mediated subunit contacts in mechanosensory transduction
Source: Proc Natl Acad Sci U S A. 2024 Feb 14;121(8):e2314096121. doi: 10.1073/pnas.2314096121 (PMC10895266; doi:10.1073/pnas.2314096121)
Supplement: Supplementary file 1 — Appendix 01 (PDF) [file pnas.2314096121.sapp.pdf]

PNAS

Supporting Information for

Structure of *C. elegans* TMC-2 complex suggests roles of lipid-mediated subunit contacts in mechanosensory transduction

Sarah Clark[1*], Hanbin Jeong[1*], Rich Posert[1], April Goehring[1,2], and Eric Gouaux[1,2]

1. Vollum Institute, Oregon Health & Science University, Portland, Oregon 97239, USA.

2. Howard Hughes Medical Institute, Oregon Health & Science University, Portland, Oregon 97239, USA.

* These authors contributed equally

Correspondence to Eric Gouaux

Email: gouauxe@ohsu.edu

This PDF file includes:

Figures S1 to S6
Table S1
SI References

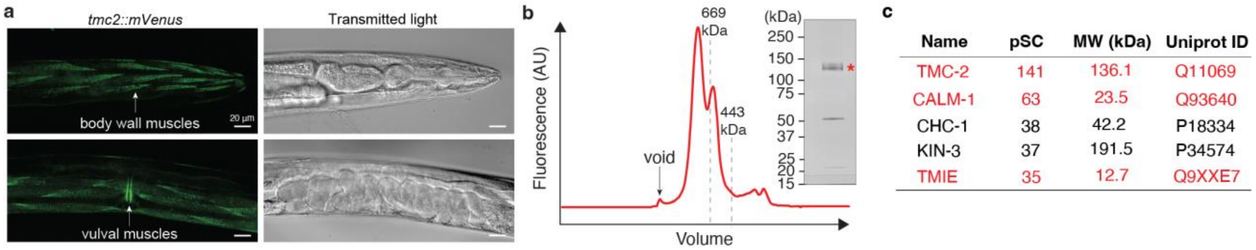

Fig. S1: Isolation of TMC-2 from *C. elegans*. a, Spectral confocal images of mVenus fluorescence and transmitted light in a *tmc2::mVenus* worm showing fluorescence in the body wall and vulval muscles. **b,** Representative FSEC profile of the TMC-2 complex, detected via mVenus fluorescence. Inset shows a silver stained SDS-PAGE gel of the purified TMC-2 complex. Red asterisk indicates TMC-2. **c,** Mass spectrometry analysis of the purified TMC-2 complex, showing the five proteins with the highest peptide spectral counts (pSC). Components of the TMC-2 complex that were also identified in the TMC-2 single particle cryo-EM structure are highlighted in red.

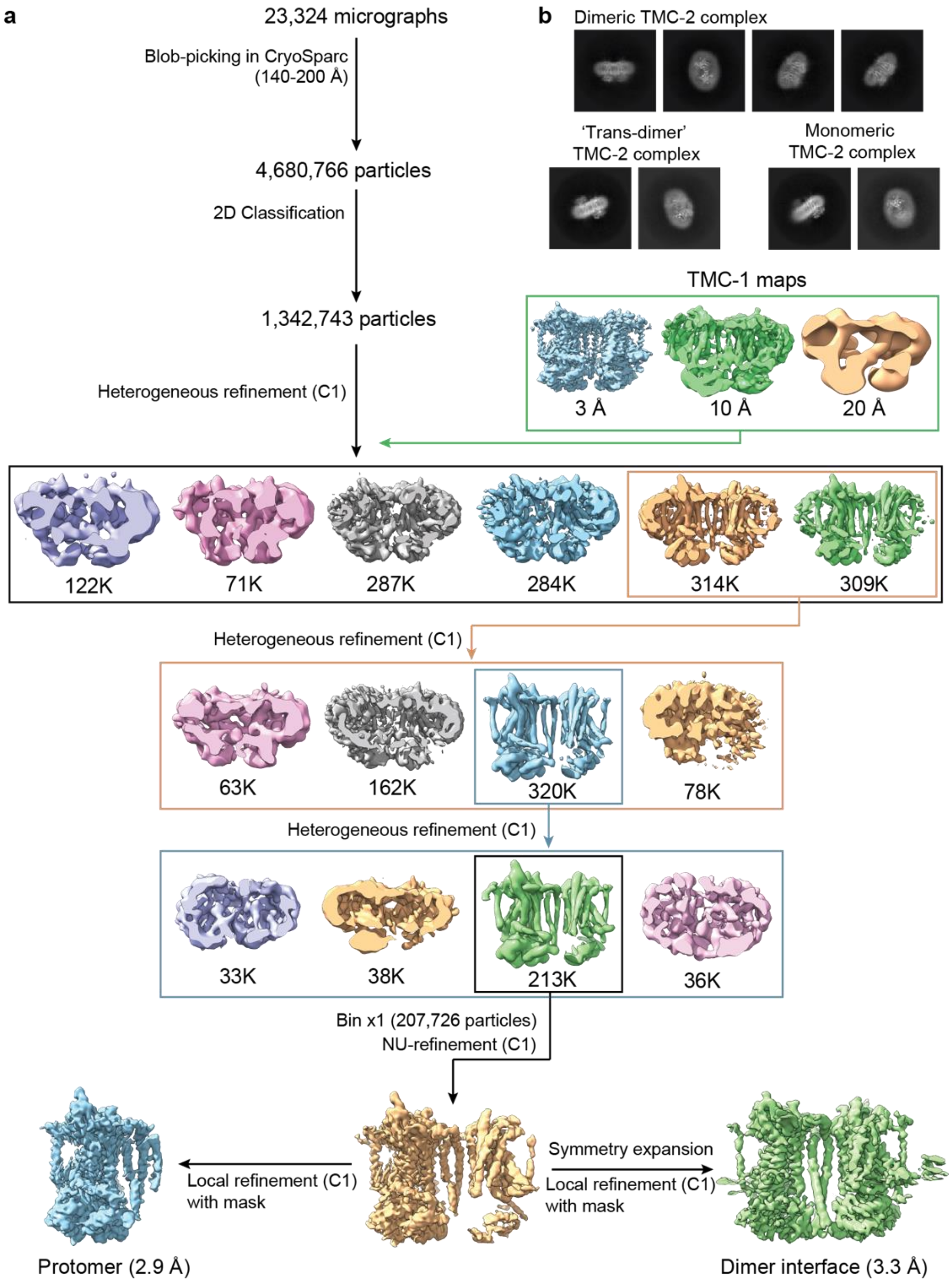

Fig. S2: Cryo-EM data analysis of the TMC-2 complex. a, Flow chart of cryo-EM data analysis of the TMC-2 complex. **b,** Representative 2D classes of the dimer TMC-2 complex (top) as well as the 'trans-dimer' and monomeric classes, the latter two of which were discarded during heterogeneous refinement.

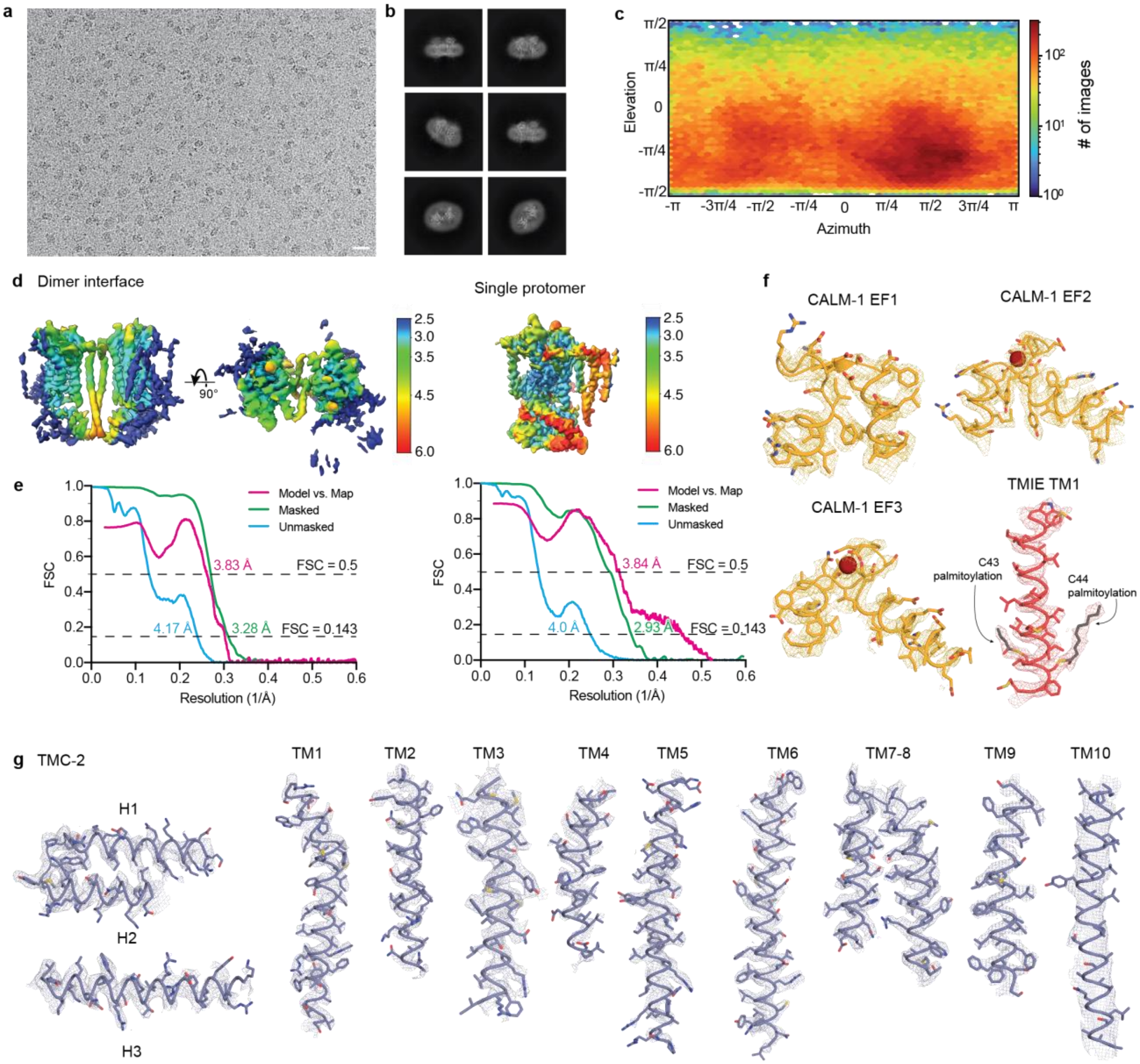

Fig. S3: Cryo-EM classes, statistics, angular distributions and selected sections of density maps. **a,** A representative cryo-EM micrograph of the TMC-2 particles. Scale bar = 200 Å. **b,** Selected 2D classes of the TMC-2 complex. **c,** Angular distributions of the final reconstruction. **d,** Density maps colored by local resolution values. **e,** Fourier shell correlations (FSEC) curves for each map and model. **f,** Fragments of cryo-EM density map and atomic models of the auxiliary subunits, CALM-1 and TMIE. Cryo-EM maps are shown as yellow and red mesh. **g,** Fragments of cryo-EM density map and atomic models of TMC-2. Cryo-EM maps are shown as purple mesh.

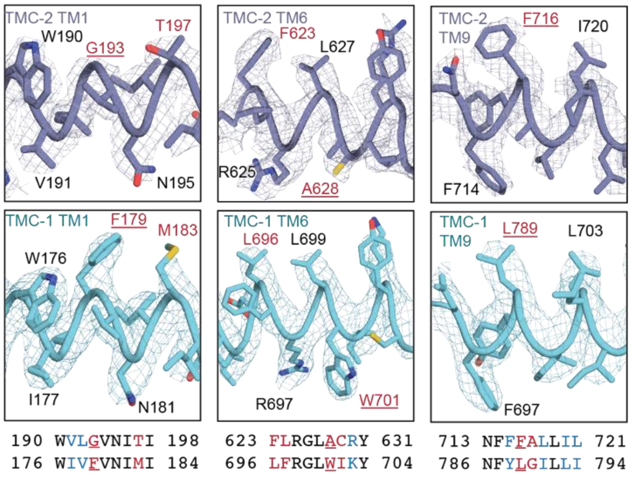

Fig. S4: Density feature differences between the TMC-1 and TMC-2 maps. The TMC-2 map and model are shown in purple and the TMC-1 map and model are shown in cyan. A sequence alignment for each fragment is shown at the bottom of the panel with the same coloring as in panel (d) of Figure 2. Underlined residues are non-conservative substitutions that are clearly distinguished in the density maps. The sequence of TMC-2 is on top and the sequence of TMC-1 is on bottom.

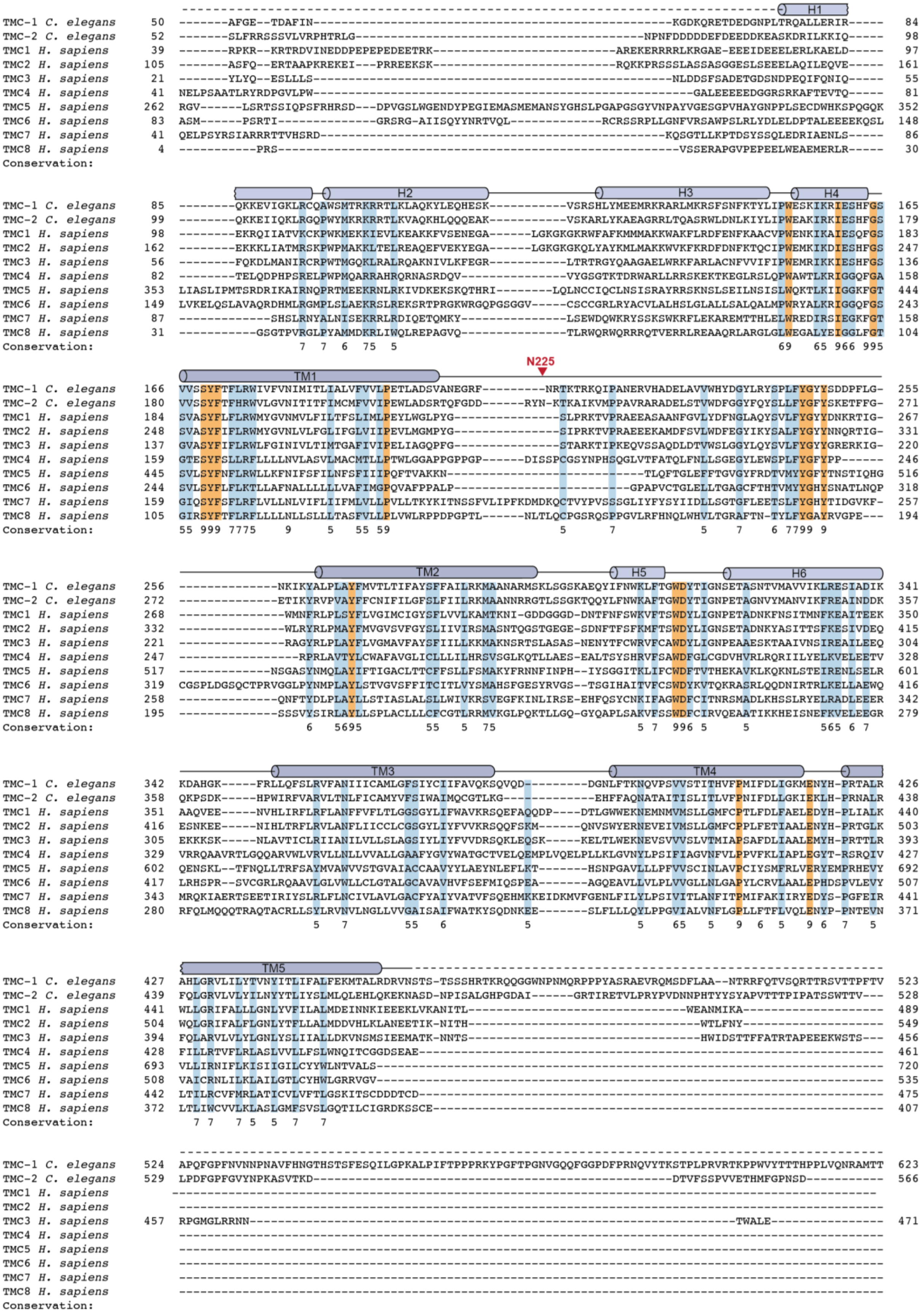
H1
H2
H3
H4
TM1
N225
TM2
H5
H6
TM3
TM4
TM5
TMC-1 C. elegans
TMC-2 C. elegans
TMC1 H. sapiens
TMC2 H. sapiens
TMC3 H. sapiens
TMC4 H. sapiens
TMC5 H. sapiens
TMC6 H. sapiens
TMC7 H. sapiens
TMC8 H. sapiens
Conservation:

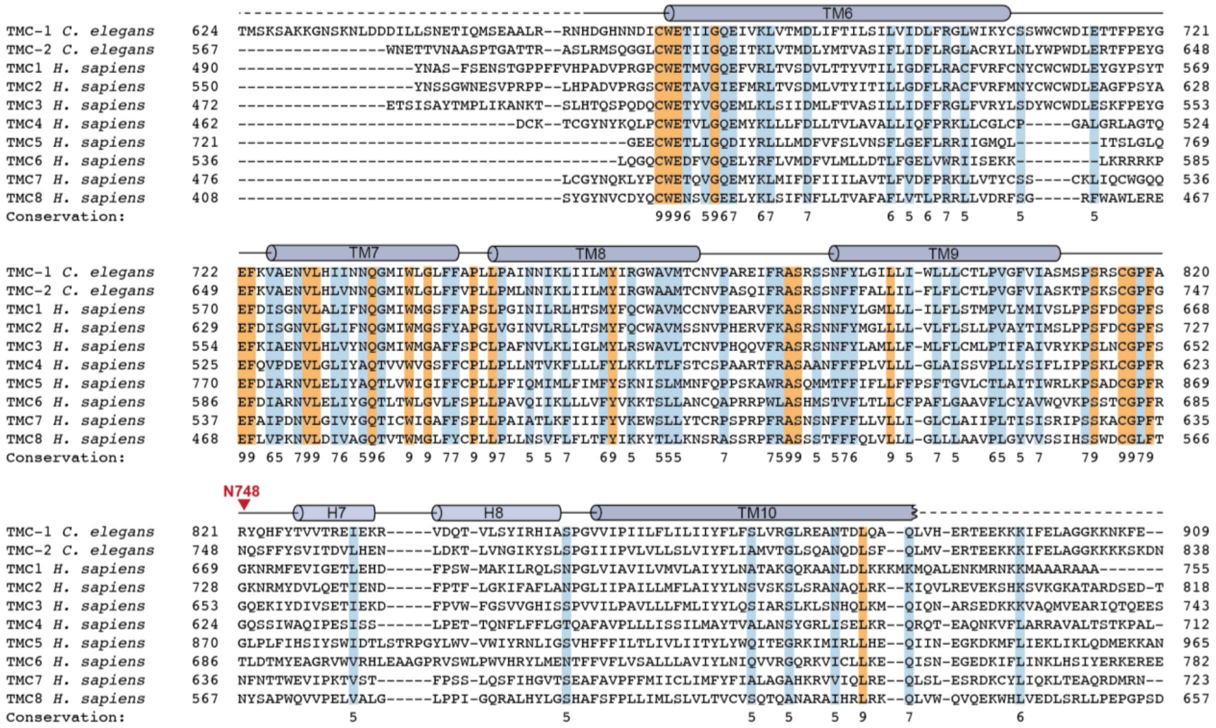

Fig. S5: Sequence alignment of TMC proteins from *C. elegans* and *H. sapiens*. Amino acid sequences from *C. elegans* TMC-1 and TMC-2 are aligned with sequences from human TMC1-8 using the program Promals3D (1). The secondary structure, based on the cryo-EM structure of *C. elegans* TMC-2, is indicated above the sequence as cylinders (α-helices), black lines (loop regions), or dashed lines (regions not visible in the structure). Conserved residues (conservation score of 9) are highlighted in orange and residues with a conservative substitution (conservation score of 5-7) are highlighted in blue. Red arrows indicate glycosylation sites in *C. elegans* TMC-2.

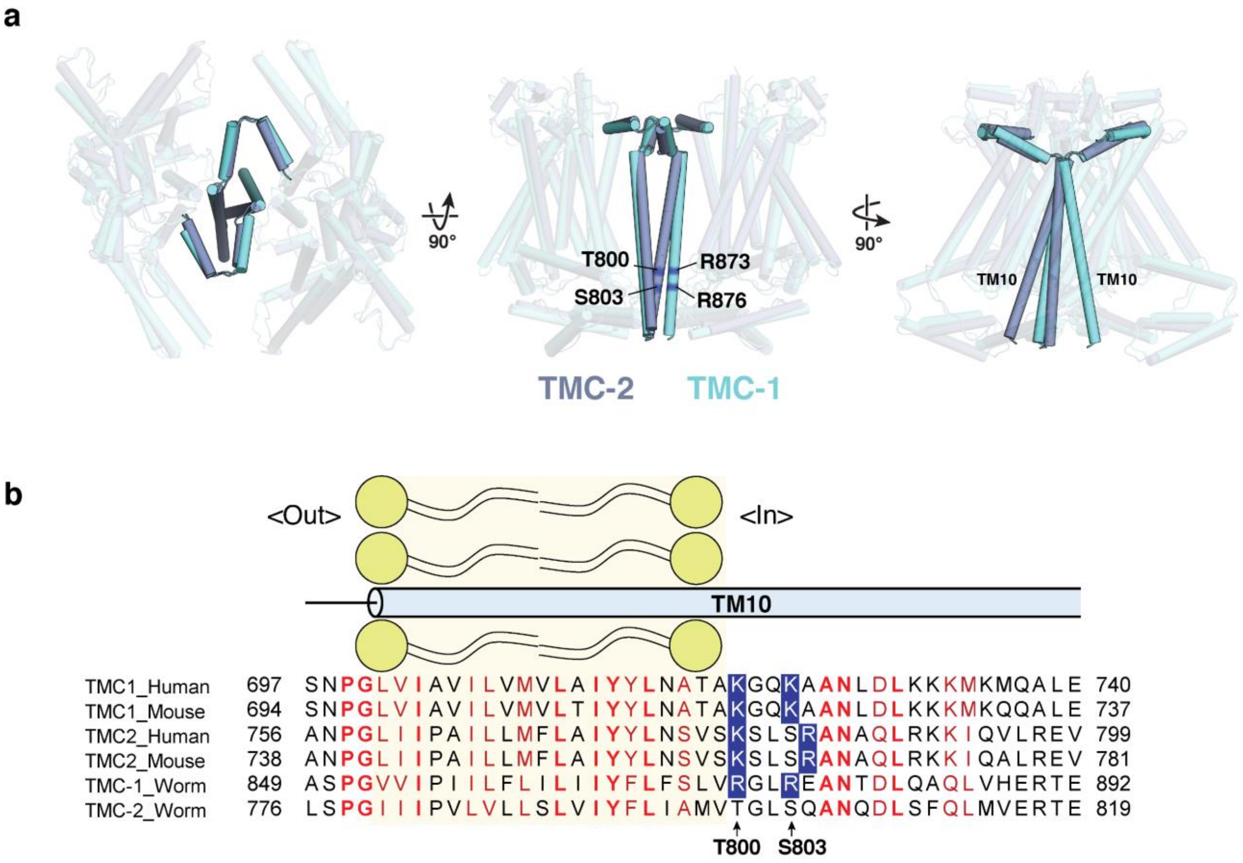

Fig. S6: TMC-1 and TMC-2 exhibit differences in their conformations and sequences at the dimer interface. a, View of the dimer interfaces, shown from three perspectives, following superposition using backbone atoms. TMC-1 is colored cyan and TMC-2 is colored purple. Basic amino acids that are proximal to the inner membrane leaflet are labeled and highlighted in blue. **b,** Multiple sequence alignment of TM10 from TMC-1 and TMC-2 from *C. elegans*, humans, and mice. Residues shown in black are not conserved, those in red are conservatively substituted, and those in bold red are fully conserved. Positively charged residues proximal to the membrane are highlighted in blue. Non-conserved residues in *C. elegans* TMC-2 are marked with black arrows. A schematic representation of TM10, showing the region that is associated with the membrane, is shown above the sequence alignment.

Table S1. Statistics for 3D reconstruction and model building

Codes	PDB 8TKP<br>Protomer: EMD-41356<br>Dimer interface: EMD-41432
**Data collection and processing**	
Microscope	Titan Krios
Camera	K3 BioQuantum
Magnification	105,000
Voltage (kV)	300
Defocus range (μm)	-1.5 to -2.5
Exposure time (s)	5.19
Dose rate (e^-/Å^2/s)	7.98
Number of frames	50
Pixel size (Å)	0.831 (0.4155 super-resolution)
Micrographs (no.)	23,364
Symmetry imposed	C1
Initial particles (no.)	4,680,766
Final particles (no.)	207,726
Map resolution (Å)	2.9 (protomer), 3.3 (dimer interface)
FSC threshold	0.143
**Refinement**	
Initial model (PDB code)	Alphafold, 7USW
Model resolution (Å)	3.2
FSC threshold	0.5
Model composition	
Non-hydrogen atoms	14666
Protein atoms	13848
Ligand atoms	818
B factors (Å^2)	
Protein	45.38
Lipids and calcium	66.06
R.m.s. deviations	
Bond length (Å)	0.002
Bond angle (˚)	0.540
**Validation**	
Favored (%)	98.04
Allowed (%)	1.96
Disallowed (%)	0
Poor rotamers (%)	1.07
MolProbity score	1.34
Clash score	5.79

SI References

1. J. Pei, N. V. Grishin, PROMALS3D: multiple protein sequence alignment enhanced with evolutionary and three-dimensional structural information. *Methods Mol Biol* **1079**, 263-271 (2014).
